# A retrospective study of demographic parameters and major health referrals among Afghan refugees in Iran

**DOI:** 10.1186/1475-9276-11-82

**Published:** 2012-12-20

**Authors:** Salman Otoukesh, Mona Mojtahedzadeh, Dean Sherzai, Arash Behazin, Shahrzad Bazargan-Hejazi, Mohsen Bazargan

**Affiliations:** 1 Department of Research, Charles R. Drew University of Medicine and science, 1731 E. 120th street, Bldg. N, Los Angeles, CA 90059, USA; 2 Department of Neurology, Loma Linda University, 11370 Anderson Street, Loma Linda, CA, 92350, USA; 3 UNHCR, Department of Field Unit, No 3, East Emdad St, Mollasadra Ave, Tehran, 19917, Iran; 4 Department of Medical Education Program, Charles R. Drew University of Medicine and science, 1731 E. 120th street, Los Angeles, CA, 90059, USA; 5 Department of Research, Charles R. Drew University of Medicine and science, 1731 E. 120th street, Bldg. N, Los Angeles, CA, 90059, USA

**Keywords:** Refugee, Health status, Ethnicity

## Abstract

**Introduction:**

For nearly three decades, the two neighboring countries of Iran and Pakistan hosted millions of Afghans. Today, Afghans still represent the largest group of refugees in the world. This feature has greatly influenced provision of health care for this population. Due to a paucity of research on the health status of Afghan refugees in Iran, this study aim to make a vista on the pattern of different common diseases among Afghan refugees in Iran and use it as an index for performance evaluation of future health services to them.

**Methods:**

This is a retrospective cross sectional study, in which we collected the demographic and medical data between 2005 and 2010 from referrals to the United Nations High Commissioner for Refugees (UNHCR) offices in Iran. We also considered a comparative review of the burden of disease estimates by the World Health Organization (WHO) for Afghanistan and Iran.

**Results:**

Total numbers of referrals were 23,152 with 52.6% Female and 47.66% male. 29% were 0–14 years of age, 54% were 15–59, and 17% were 60+. The most common health referral for females and males (0–14) was perinatal diseases (15.16%, 15.2%, respectively). In the females (15–59) it was ophthalmic diseases (13.65%), and for males it was nephropathies (21.4%), and in both sexes (60+) age range it was ophthalmic diseases (21.3%, 19.9%, respectively). The largest ethnic group of afghan refugees in this study was Hazara (55%) followed by Tajik (14%), Fars (12%), Sadat (9%), and 10% others. Ophthalmic diseases were the major cause of referrals by Hazara, Tajik, Fars, and Sadat groups with 26%, 20%, 26%, and 27% respectively. Referrals by pashtun group were mostly for neoplasms (17%), among Uzbek group it was nephropathies (26%), and in Baluch group Hematopoietic disorders (25%).

**Conclusion:**

These data indicate higher referral rate for women 15–59 years of old and people in 60+ with ophthalmic diseases, neoplasms, and nephropathies. Even given certain intrinsic limitations of such a study, we believe these unique findings are worth further explanation. This implies the need for public health researchers to pursue prospective studies in these areas.

## Introduction

Afghanistan’s domestic upheaval in the 1970’s, followed by the Soviet occupation in 1979, resulted in a massive displacement of its population. After the Soviet withdrawal in 1989 and the removal of the communist regime in 1992, about three million Afghans returned from exile. However, neither the rise of the Taliban in 1994, nor their fall in 2001, mitigated the challenges facing the diaspora who fled the country in different waves, and have had to maintain their immigrant status.

Years of conflict have inflicted near fatal wounds upon the healthcare infrastructure throughout Afghanistan. According to the Ministry of Public Health (2009), the country has suffered a devastating decline during the past three decades, with human and socio-economic indicators hovering near the bottom of international indices [1]. For nearly three decades, the neighboring countries of Iran and Pakistan have hosted millions of Afghans. Afghans today in fact represent the largest group of refugees in the world [2]. The Afghan situation in Iran is characterized by a) prolonged exile, b) large numbers (1,019,700 Afghan refugees as of July 2011), c) residence in urban areas, d) the emergence of a second generation, and e) a significant social support system provided by the host country [3-5]. These factors have largely shaped and determined healthcare for this population.

There is, however, a paucity of data on the health status of Afghans in exile. Previous studies were limited in scope in regards to sampling, or in areas of coverage. One clinic for Afghan refugees in Pakistan reported that most referrals were for gastrointestinal tract disorders, followed by respiratory tract complaints [6]. One research project in Northern Pakistan focused on the prevalence and etiology of visual loss and eye diseases in a resident Afghan refugee community [7]. A number of other studies have addressed tuberculosis (TB) and the mental health problems of Afghan refugees in Iran [8-15], Pakistan [16,17], the United States [18-21] and the Netherlands [22,23]. The scarcity of health data is also a challenge in Afghanistan. According to the Afghan Ministry of Public Health (2011), minimal data exists on the current health status of the population and on resource allocations in the health sector [24].

The World Health Organization (WHO) has stated that Afghanistan is a country where there is limited knowledge on most causes of mortality and morbidity [25]. Afghanistan’s non-communicable diseases (NCD) country profile, as reported by WHO (2011), reveals that mortality estimates have a high degree of uncertainty because they are not based on any national NCD mortality data. These estimates are based on a combination of country life tables, cause of death models, regional cause of death patterns, and WHO and UNAID program estimates for some major causes of death. These do not include NCDs, which are estimated to account for 29% of all deaths in the WHO report [26]. Due to a paucity of research on the health status of Afghan refugees in Iran, this study aims to illustrate patterns of common diseases among Afghan refugees in Iran, and to use these data as an index for evaluating the performance of future health services.

## Methods

This is a retrospective cross-sectional study that uses data collected from the UNHCR offices in Tehran and Mashhad. This database represents approximately 85% of Afghan refugees registered in Iran. These data were collected by two teams, each of which included at least three professional social workers and interviewers, one physician and one general purpose receptionist. These personnel, based in the UNHCR offices, held responsibility for extended areas in the central, northern and eastern parts of Iran.

Each case or patient approached UNHCR Offices for assistance, or were referred by hospitals, welfare, charity societies, or governmental and non-governmental organizations. Requests for assistance were screened and processed by the community and medical service teams through interviews conducted at home, in hospitals and in community visits. A report was recorded in the Community Integrated Social and Medical Assistance Program (CISAMAP) database by the interviewer after each case had given his or her consent. Records include all accepted and rejected cases for assistance, and are based on a list of referral causes (Additional file: 1) to ensure reliable data entry. Medical assessment of each case rested solely with the CISAMAP physician.

Data extracted for this study is based on a sample of 23,167 registered Afghan refugees who were referred from 2005 to 2010, a six year period. Tables and graphs represent disaggregated data for age, gender and ethnicity of Afghan refugees for inter- and intra-group comparisons. This information was reviewed along with the Global Burden of Disease 2011 Report released by WHO. SPSS (version 18) was used initially for data analysis; tables and graphs were prepared in Microsoft Word 2010.

### Findings

The total number of cases in this study was 23,152. Most referrals were for females (52.6%), followed by males (47.66%) The most frequent causes for referrals were for ophthalmic diseases (23.7%), neoplasm (13.3%), nephropathies (11%), ischemic heart diseases (10.4%), and perinatal disorders (9.2%) (Additional file: 1). The Hazara represented the largest ethnic group of Afghan refugees (40.47%), followed by the Tajik (22%), Pashtun (8.8%), Sadat (6.6%), Fars (5.25%), Baluch (3.1%), and Uzbek (2.51%). By age, 38% of Afghan refugees in Iran are 0 – 14, 59% are 15 – 59, and 3% are 60+ [27], with the percentage of referrals to UNHCR being 29%, 54%, and 17% for each of these groups, respectively. The highest referral rate was for females 60+ years of age (17%); for every 100 women age 60 and older, 17 received referrals. The rate for men in the same age group was 0.13 (Table 1).

**Table 1:** Referral rates

	**0-14**			**15-59**			**60+**			**Total**
	**Male**	**Female**	**All**	**Male**	**Female**	**All**	**Male**	**Female**	**All**	
**Total in the country**	199397	186027	385424	329384	271050	600434	21748	13717	35465	**1021323**
**Respective provinces**	150026	139967	289993	247829	203938	451767	16363	10321	26684	**768444**
**Referrals**	3449	3261	6710	5503	7082	12585	2081	1776	3857	**23152**
Referral rates	**0.02**	**0.02**	**0.02**	**0.02**	**0.03**	**0.03**	**0.13**	**0.17**	**0.14**	**0.03**

Sources: Amayesh 2005 for total in the country and respective provinces (estimated) and UNHCR Database 2005–2010 for referral.

### Cause of referrals for different age groups

Prenatal disorders (30%), ophthalmic diseases (21%), and congenital anomalies (15%) made up about 66% of referrals for patients 0–14 years of age. Ophthalmic diseases, nephropathies, neoplasm, and ischemic heart diseases were the most common cause of referrals for those 15–59 years of age (total 64%), at 20%, 16%, 16%, and 12% respectively. Ophthalmic diseases, ischemic heart disease, and neoplasm constituted 74% of referrals for those 60 or older, at 41%, 21%, and 12% respectively Table 2.

**Table 2:** Causes of referrals by age and gender distribution

**Cause of referrals**		**0_14**			**15_59**			**60+**			**Total**
		**F**	**M**	**All**	**F**	**M**	**All**	**F**	**M**	**All**	
**Ophtalmic disease**	Count	80	580	**1385**	1591	790	**2508**	822	769	**1591**	5484
	%	24.70%	16.80%	**20.60%**	24.30%	14.40%	**19.90%**	46.30%	37.00%	**41.20%**	23.70%
**Neoplasms**	Count	267	327	**594**	1143	870	**2013**	164	312	**476**	3083
	%	8.20%	9.50%	**8.90%**	16.10%	15.80%	**16.00%**	9.20%	15.005	**12.30%**	13.30%
**Nephropathies**	Count	105	128	**233**	859	1181	**2040**	87	188	**275**	2548
	%	3.205	3.70%	**3.505**	12.10%	21.50%	**16.20%**	4.90%	9.00%	**7.10%**	11.00%
**Ischemic Heart Disease**	Count	17	17	**34**	768	794	**1562**	389	412	**801**	2397
	%	0.50%	0.50%	**0.50%**	10.80%	14.40%	**12.40%**	21.90%	19.80%	**20.80%**	10.40%
**Perinatal disease**	Count	1017	1020	**2037**	57	36	**93**	2	0	**2**	2132
	%	31.20%	29.60%	**30.40%**	0.80%	0.70%	**0.70%**	0.10%	0.00%	**0.10%**	9.20%
**Congenital anomalies**	Count	485	531	**1016**	80	66	**146**	0	0	**0**	1162
	%	14.90%	15.40%	**15.10%**	1.10%	1.20%	**1.20%**	0.00%	0.00%	**0.00%**	5.00%
**Appendicitis**	Count	86	119	**205**	370	461	**831**	6	7	**13**	1049
	%	2.60%	3.50%	**3.10%**	5.20%	8.40	**6.60%**	0.30%	0.30%	**0.30%**	4.50%
**Labor complications**	Count	4	2	**6**	754	5	**759**	1	0	**1**	766
	%	0.10%	0.10%	**0.10%**	10.60	0.10%	**6.00**	0.10%	0.00%	**0.00%**	3.30%
**Deafness**	Count	117	93	**210**	199	139	**338**	50	98	**148**	696
	%	3.60%	2.70%	**3.10%**	2.80%	2.50%	**2.70%**	2.805	4.70%	**3.80%**	3.00%
**Liver, Billiary, Pancreas disease**	Count	14	20	**34**	381	134	**515**	72	72	**144**	693
	%	0.40%	0.60%	**0.50%**	5.40%	2.40%	**4.10%**	4.10%	3.50%	**3.70%**	3.00%
**Urinary disorders**	Count	43	59	**102**	201	251	**452**	29	62	**91**	645
	%	1.30%	1.70%	**1.50%**	2.80%	4.60%	**3.60%**	1.60%	3.00%	**2.40%**	2.80%
**Neurologic disorder**	Count	144	190	**334**	81	144	**225**	37	40	**77**	636
	%	4.40%	5.505	**5.00%**	1.10%	2.60%	**1.80%**	2.10%	1.90%	**2.00%**	2.70%
**Fractures**	Count	30	77	**107**	91	372	**463**	25	37	**62**	632
	%	0.90%	2.20%	**1.60%**	1.30%	6.80%	**3.70%**	1.40%	1.80%	**1.60%**	2.70
**Hematologic diseases**	Count	96	265	**361**	117	137	**254**	3	6	**9**	624
	%	2.90%	7.70%	**5.40%**	1.70%	2.50%	**2.00%**	0.20%	0.30%	**0.20%**	2.70%
**TB (all forms**	Count	31	21	**52**	263	123	**386**	89	78	**167**	605
	%	1.00%	0.605	**0.80%**	3.70%	2.20%	**3.10%**	5.00%	3.70%	**4.30%**	2.60%
**Total**	%	3261	3449	**6710**	7082	5503	**12585**	1776	2081	**3857**	23152
		**100.00%**	**100.00%**	**100.00%**	**100.00%**	**100.00%**	**100.00%**	**100.00%**	**100.00%**	**100.00%**	**100.00%**

### Cause of referrals by gender

In our study, 12,126 females (52.34%) and 11,041 males (47.66%) received referrals. Although more females received referrals than males, they shared the most common causes, including ophthalmic diseases (9.2% vs. 14.4%), neoplasm (6.5% vs. 6.8%), nephropathies (6.4% vs. 4.5%), and ischemic heart disease (5.2% vs. 5%).

### Cause of referrals by ethnicity

By ethnicity, the Hazara received the highest number of referrals (55%), followed by the Tajik at 14%, Fars at 11%, Sadat at 8%, Pashtun at 2%, and Uzbek at 1%. Ophthalmic diseases were the major cause of referrals with the Hazara, Tajik, Fars and Sadat at 26%, 20%, 26%, and 27% respectively. The disproportionate frequency of referrals among the Pashtun for neoplasmic disease (17%) is noteworthy, as with nephropathies among the Uzbek at 26%, and hematopoietic disorders (25%) for the Baluch Table 3.

**Table 3:** Cause of referrals by ethnicity distribution

**Disease**		**BALOCH**	**FARS**	**HAZARA**	**PASHTUN**	**SADAT**	**TAJIKA**	**UZBEK**	**OTHERS**	**NOT INDICATED**	**TOTAL**
**Ophtalmic disease**	Count	7	682	3310	58	490	635	17	157	129	5485
	%	9.2	26.2	25.9	10.2	27.3	19.6	12.4	11.8	21.3	23.7
**Neoplasma**	Count	16	290	1646	99	172	493	17	242	109	3084
	%	21.1	11.1	12.9	17.3	9.6	15.2	12.4	18.1	18	13.3
**Nephropathies**	Count	6	158	1461	61	219	371	35	188	49	2548
	%	1.9	6.1	11.4	10.7	12.2	11.4	25.5	14.1	8.1	11
**Ischemic Heart Disease**	Count	5	437	1150	51	165	375	4	146	64	2397
	%	6.6	16.8	9	8.9	9.2	11.6	2.9	10.9	10.5	10.3
**Perinatal disease**	Count	1	203	1140	72	188	305	10	134	85	2138
	%	1.3	7.8	8.9	12.6	10.5	9.4	7.3	10	14	9.2
**Congenital anomalies**	Count	4	38	644	52	86	182	19	95	48	1168
	%	5.3	1.5	5	9.1	4.8	5.6	13.9	7.1	7.9	5
**Appendicitis**	Count	1	206	534	10	74	162	0	45	17	1049
	%	1.3	7.94	4.2	1.8	4.1	5		3.4	2.8	4.5
**Labor complications**	Count	0	116	432	4	49	137	1	20	7	766
	%		4.5	3.4	0.7	2.7	4.2	0.7	1.5	1.2	3.3
**Deafness**	Count	1	137	384	2	70	83	0	15	4	696
	%	1.3	5.3	3	0.4	3.9	2.6		1.1	0.7	3
**Liver, Billiary, Pancreas disease**	Count	8	15	423	34	62	84	8	30	29	693
	%	10.5	0.6	3.3	6	3.5	2.6	5.8	2.2	4.8	3
**Urinary disorders**	Count	3	136	321	13	51	90	0	24	7	645
	%	3.9	5.2	2.5	2.3	2.8	2.8		1.8	1.2	2.8
**Neurologic disorder**	Count	2	143	317	6	53	78	1	30	6	636
	%	2.6	5.5	2.5	1.1	3	2.4	0.7	2.2	1	2.7
**Fracture**	Count	3	18	399	20	43	81	14	37	17	632
	%	3.9	0.7	3.1	3.5	2.4	2.5	10.2	2.8	2.8	2.7
**Hematologic diseases**	Count	19	10	213	63	35	108	5	155	17	625
	%	25	0.4	1.	11	1.9	3.3	3.6	11.6	2.8	2.7
**TB (all forms**	Count	0	15	426	26	39	57	6	17	19	605
	%		0.6	3.3	4.6	2.2	1.8	4.4	1.3	3.1	2.6
**Total**	%	100%	100%	100%	100%	100%	100%	100%	100%	100%	100%

Source: UNHCR Database 2005-2010.

## Discussion

The tremendous uncertainty that surrounds the health status of millions of refugees in exile underscores the need for health referral data for this population. Currently, the worldwide occurrence of non-communicable disease is 43%, but is expected to increase to 60% and cause 73% of all deaths by 2020. Most of this will occur through epidemics in developing countries such as Iran, especially among refugee populations [28-31].

### Age and gender

Afghanistan is in the early stages of demographic transition, which will become more evident by 2025 [28,32]. The percentage of the population 65 years of age and older will increase from 2.1% in 2000 to 2.9% in 2025 [32]. Older residents are more likely to be affected by NCDs, and it is expected that disease rates will rise commensurate with aging [32].

The most common cause of referrals among 0–14 year olds was perinatal disorders, which are documented as communicable diseases in Afghanistan and Iran. In this age group, referral rates for males and females are identical. Better health status, along with greater access to health services in Iran, are thought to have resulted in reduced referral rate for refugees in this age group, compared to similar populations in Afghanistan. However, because medical costs are higher for refugees compared with citizens in Iran, limitations may eventually restrict access.

Those15-59 years of age had 54% of referrals and constitute the largest number of Afghan refugees. Ophthalmic diseases were the most common cause of referrals. Because this age group represents the bulk of the workforce in the diaspora, the impact of these diseases is clear. Referral rates in this group were higher for females. This can be attributed to the role of women as the head of the household, as well as to the documented reluctance of men to seek medical care due to the high cost [33].

In our study, only 17% of referrals were by refugees aged 60+. The most common condition was ophthalmic, followed by cardiovascular disease. Referral rates were higher for women in this age group, which may be attributed to two factors. Afghan women in Iran have been traditionally involved in handicraft, which has been associated with greater occurrences of ophthalmic diseases [34,35]. In refugee settings, men are also seen to use health care services less frequently than women [33].

Chronic diseases such as heart disease and stroke are prevalent among elderly populations, including refugees [36,37]. However, the reduced number of chronic cases in our population may be attributed to factors such as a) language barriers and incorrect interpretation and translation services [38], b) cultural and structural barriers [39], and c) the lack of access for preventative care and treatment [40].

### Ethnicity

The Hazara, Tajik, Fars and Sadat ethnic groups incurred the most referrals for ophthalmic diseases, probably as a result of their trade and livelihood, e.g. construction workers, handicraft jobs [34,35]. The number of referrals for smaller groups such as the Pashtun and Baluch may not be truly representative, as they reside mainly in the south and southeast of Iran, and data for these populations may be incomplete.

Afghan refugees are uniquely distributed in neighboring countries for several reasons. With the communist takeover of 1978, their migration has been heterogeneous in regards to race and religion. History, culture and religious differences have had a significant impact on where Afghans have settled. Pashtuns have more often migrated to Pakistan because of ethnic, linguistic and religious similarities. Nearly 40 million Pakistanis in the region bordering Afghanistan are of Pashtun origin, speak Pashtu and are Sunni Muslims, germane to their Afghan refugee counterparts. The Hazara are mostly Shiite, speak Farsi, and live mainly in the northern and northeastern regions of Afghanistan. This religious and linguistic proximity draws them disproportionately to Iran (55% vs. 40.47% of all refugees) [41].

### Limitations

The retrospective analysis of data from UNHCR offices in Iran limits our choice of variables, and may be inferior to a prospective, active data collection research paradigm. Most retrospective studies rely on the accuracy of data records, and/or the recollection of individuals. Similarly, our study has relied on the accuracy of data entry by interviewers. Moreover, inconsistencies in record keeping between UNHCR offices did not allow comprehensive data to be compiled for the entire country of Iran. Referral rates were calculated assuming equal access to referrals by all Afghan refugees, and on the homogeneous distribution of age groups in the country.

There are more than 2 million unregistered foreigners in Iran, mostly Afghan and Iraqi nationals, who were not included in this study [3]. This report also does not consider communicable, diarrheal and parasitic diseases which are prevalent in Afghanistan and are considered a major part of the healthcare burden in Iran [42]. These conditions may be mitigated by access to safe drinking water and vaccination, and allocation of resources to costly in-patient treatment and care.

## Conclusions

Our study is unique in that it provides a comprehensive look into perhaps the largest diaspora of Afghans. Important findings include that, for those 0–14 years of age, prenatal disease was the most common cause in seeking healthcare, whereas those 15–59 and > 60 years of age were referred primarily for ophthalmic diseases, neoplasms, and nephropathies. We also highlight differences in disease proclivity by ethnicity, which may facilitate better access and effective treatment. Despite the intrinsic limitations inherent in such a study, these findings promise to provide insight into providing improved access and care for this beleaguered population.

## Competing interests

The authors declare that they have no competing interests.

## Authors' contributions

SO, MM, AB and D.SH were involved in the study from the design to the final manuscript. MM, MB and SH.B-H analyzed the data. AB drafted the first manuscript. SO, D.SH and MB reviewed the paper. All authors have read and approved the final manuscript.

## Supplementary Material

Additional file 1 **Appendix1.** Cause of referrals. Click here for file
