# Supplementary material for: A retrospective study of demographic parameters and major health referrals among Afghan refugees in Iran
Source: Int J Equity Health. 2012 Dec 20;11:82. doi: 10.1186/1475-9276-11-82 (PMC3547741; doi:10.1186/1475-9276-11-82)
Supplement: Additional file 1 — Appendix1. Cause of referrals. [file 1475-9276-11-82-S1.docx]

***Appendix 1- cause of referrals***

| Type of Referrals (%) | Number of referrals (%) |
| --- | --- |
| ***Ophthalmic Diseases (23.7)*** | ***5485*** |
| 1. Retinal detachments and defects | **139 (0.6)** |
| 1. Glaucoma | **185 (0.8)** |
| 1. Cataract | **2569 (11.1)** |
| 1. Disorder of refraction and accommodation | **2152 (9.3)** |
| 1. Keratitis | **93 (0.4)** |
| 1. Corneal Opacity and other disorder of cornea | **69 (0.3)** |
| 1. Disorders of optic nerve and visual pathways | **254 (1.1)** |
| 1. Strabismus and other disorders of binuclear eye movements | **24 (0.1)** |
| ***Neoplasms (13.3)*** | ***3084*** |
| 1. Malignant neoplasm of lip, oral cavity, and pharynx | **71 (0.3)** |
| 1. Malignant neoplasm of digestive organs and peritoneum | **1008 (4.3)** |
| 1. Malignant neoplasm of respiratory and intra thoracic organs | **136 (0.6)** |
| 1. Malignant neoplasm of bone, connective tissues and skin | **360 (1.6)** |
| 1. Malignant neoplasm of genitourinary organs | **263 (1.1)** |
| 1. Malignant neoplasm of other sites such as brain, eye, ear, … | **308 (1.3)** |
| 1. Malignant neoplasm of lymphatic and hematopoietic tissue | **640 (2.9)** |
| 1. Benign neoplasm | **141 (0.6)** |
| 1. Carcinoma in situ | **6 (0.01)** |
| 1. Malignant neoplasm of female breast | **119 (0.5)** |
| 1. Malignant neoplasm of thyroid gland | **25 (0.1)** |
| 1. Malignant neoplasm of other endocrine glands | **7 (0.01)** |
| ***Nephropathies (11)*** | ***2548*** |
| 1. Acute glomerulonephritis | **93 (0.3)** |
| 1. Nephrotic Syndrome | **133 (0.6)** |
| 1. Chronic glomerulonephritis | **23 (0.1)** |
| 1. Nephritis and nephropathy not specified acute or chronic | **23 (0.1)** |
| 1. Acute renal failure | **83 (0.3)** |
| 1. Chronic renal failure (Dialysis) | **2193 (9.6)** |
| ***Ischemic Heart Disease (10.3)*** | ***2397*** |
| 1. Acute myocardial infarction | **258 (1.1)** |
| 1. Old myocardial infarction | **48 (0.2)** |
| 1. Angina pectoris | **1253(5.4)** |
| 1. Other forms of heart diseases: Acute pericarditis/Acute and sub-acute endocarditis/Acute myocarditis/Cardiomyopathy/Conduction disorders/Cardiac dysrhythmia/Hear failure | **838(3.6)** |
| ***Perinatal Disorders (9.2)*** | ***2138*** |
| 1. Slow fetal growth and fetal malnutrition | **116 (0.5)** |
| 1. Birth trauma | **25 (0.09)** |
| 1. Intrauterine hypoxia and birth asphyxia | **139 (0.6)** |
| 1. Respiratory distress syndrome | **1185 (5.1)** |
| 1. Infections specific to the perinatal period | **348 (1.5)** |
| 1. Fetal and neonatal hemorrhage | **2 (0.01)** |
| 1. Hemolytic disease of fetus or newborn due to immunization | **69 (0.3)** |
| 1. Other perinatal jaundice | **139 (0.6)** |
| 1. Hematological disorders of fetus and newborn | **46 (0.2)** |
| 1. Perinatal disorders of digestive system | **69 (0.3)** |
| ***Congenital Anomalies (5)*** | ***1168*** |
| 1. TORCH related anomalies | **46 (0.2)** |
| 1. Spina bifida | **23 (0.1)** |
| 1. Bulbus cordis anomalies of cardiac septal closure | **327 (1.4)** |
| 1. Other congenital anomalies of circulatory system | **187 (0.7)** |
| 1. Congenital anomalies of respiratory system | **23 (0.1)** |
| 1. Cleft palate and cleft lip | **93 (0.4)** |
| 1. Congenital anomalies of digestive system | **257 (1.1)** |
| 1. Congenital anomalies of genital organs | **46 (0.2)** |
| 1. Congenital anomalies of urinary system | **70 (0.3)** |
| 1. Certain congenital musculoskeletal deformities | **93 (0.4)** |
| 1. Chrosomal anomalies | **3 (0.01)** |
| ***Appendicitis (4.5)*** | ***1049*** |
| ***Labor Complication (3.6)*** | ***766*** |
| 1. Obstructed labor | **21 (0.1)** |
| 1. Long labor | **660 (3.1)** |
| 1. Umbilical cord complication | **9 (0.05)** |
| 1. Trauma to perineum and vulva during delivery | **2 (0.01)** |
| 1. Postpartum hemorrhage | **2 (0.01)** |
| 1. Retained placenta or membranes without hemorrhage | **2 (0.01)** |
| ***Deafness & Disease of the Ear and Mastoid Process (3)*** | ***787*** |
| ***Gastrointestinal Disorders (3)*** | ***745*** |
| 1. Disease of the esophagus | **4 (0.02)** |
| 1. Peptic ulcer, site unspecified | **2 (0.02)** |
| 1. Gastritis and duodenitis | **14 (0.07)** |
| 1. Hernia (abdominal cavity/ inguinal) | **115 (0.5)** |
| 1. Non infective enteritis and colitis | **2 (0.01)** |
| 1. Viral disease accompanied by exanthema | **2 (0.01)** |
| 1. Intestinal obstruction without mention of hernia | **20 (0.1)** |
| 1. Diverticulitis of Intestine | **21(0.1)** |
| 1. Anal fissure and fistula | **94 (0.4)** |
| 1. Abscess of anal and rectal region | **23 (0.1)** |
| 1. Peritonitis | **40 (0.2)** |
| 1. Acute and sub-acute necrosis of liver | **2 (0.01)** |
| 1. Chronic liver disease and cirrhosis | **99 (0.4)** |
| 1. Liver abscess and sequel of chronic liver disease | **22 (0.1)** |
| 1. Other disorders of liver | **20 (0.1)** |
| 1. Cholelithiasis | **254 (1)** |
| 1. Other disorder of billiary tract | **20 (0.1)** |
| 1. Disease of pancreas | **22 (0.1)** |
| 1. Gastrointestinal hemorrhage | **23 (0.1)** |
| ***Urinary Diseases (2.8)*** | ***645*** |
| 1. Infections of kidney | **20 (0.1)** |
| 1. Hydronephrosis | **18(0.1)** |
| 1. Calculus of kidney and ureter | **423 (1.8)** |
| 1. Calculus of lower urinary tract | **53 (0.2)** |
| 1. Cystitis | **17 (0.1)** |
| 1. Urethritis, not sexually transmitted and uretheral syndrome | **10 (0.01)** |
| 1. Urethral stricture | **40 (0.2)** |
| 1. Hyperplasia of prostate | **20 (0.1)** |
| 1. Hydrocele | **29 (0.1)** |
| 1. Orchitis and epididymitis | **7 (0.01)** |
| 1. Disorders of penis | **8 (0.01)** |
| ***Neurologic Diseases (2.7)*** | ***636*** |
| 1. Hereditory and degenerative diseases of the central nervous system | **2 (0.01)** |
| 1. Parkinson’s disease | **2 (0.01)** |
| 1. Subarachnoid Hemorrhage | **16 (0.08)** |
| 1. Intracerebral hemorrhage | **2 (0.01)** |
| 1. Late effects of cerebrovascular disease | **2 (0.01)** |
| 1. Multiple sclerosis | **23 (0.1)** |
| 1. Hemiplegia | **188 (0.8)** |
| 1. Epilepsy | **212 (0.9)** |
| 1. Migrane | **69 (0.3)** |
| 1. Hereditory and idiopathic peripheral neuropathy | **2 (0.01)** |
| 1. Inflammatory and toxic neuropathy | **2 (0.01)** |
| 1. Inflammatory disease of the central nervous system | **2 (0.01)** |
| 1. Meningitis | **92 (0.4)** |
| 1. Encephelitis & myelitis and encephalomyelitis | **2 (0.01)** |
| 1. Intracranial and intra spinal abscess | **8 (0.04)** |
| ***All type of Fractures (2.7)*** | ***632*** |
| ***Hematopoietic disorders (2.7)*** | ***625*** |
| ***Tuberculosis (2.6))*** | ***605(2.6)*** |
| 1. Pulmonary TB | **-------** |
| 1. TB of meninges and central nervous system | **-------** |
| 1. TB of intestines, peritoneum and mesenteric vessels | **-------** |
| 1. TB of bones and joints | **-------** |
| 1. TB of genitourinary system | **-------** |
| 1. Miliary TB | **-------** |
| 1. TB of other organs | **-------** |
| Total | 23167 |

Source: UNHCR CISAMAP Database 2005- 2010 Tehran and Mashhad
